# High-Precision Automated Workflow for Urinary Untargeted Metabolomic Epidemiology

**DOI:** 10.1021/acs.analchem.1c00203

**Published:** 2021-03-19

**Authors:** Isabel Meister, Pei Zhang, Anirban Sinha, C. Magnus Sköld, Åsa M. Wheelock, Takashi Izumi, Romanas Chaleckis, Craig E. Wheelock

**Affiliations:** † Gunma University Initiative for Advanced Research (GIAR), Gunma University, 3-39-22 Showa-machi, Maebashi, Gunma 371-8511, Japan; ‡ Division of Physiological Chemistry 2, Department of Medical Biochemistry and Biophysics, Karolinska Institutet, Biomedicum Quartier 9A, Stockholm 171-77, Sweden; § Department of Respiratory Medicine, Amsterdam UMC, University of Amsterdam, Meibergdreef 9, Amsterdam 1105 AZ, The Netherlands; ∥ Department of Experimental Immunology, Amsterdam UMC, University of Amsterdam, Meibergdreef 9, Amsterdam 1105 AZ, The Netherlands; ⊥ Computational Physiology and Biostatistics, University Children’s Hospital, Spitalstrasse 33, Basel 4056, Switzerland; # Respiratory Medicine Unit, K2 Department of Medicine Solna and Center for Molecular Medicine, Karolinska Institutet, Stockholm 141-86, Sweden; ¶ Department of Respiratory Medicine and Allergy, Karolinska University Hospital, Stockholm 141-86, Sweden; ∇ Department of Biochemistry, Gunma University Graduate School of Medicine, 3-39-22 Showa-machi, Maebashi, Gunma 371-8511, Japan

## Abstract

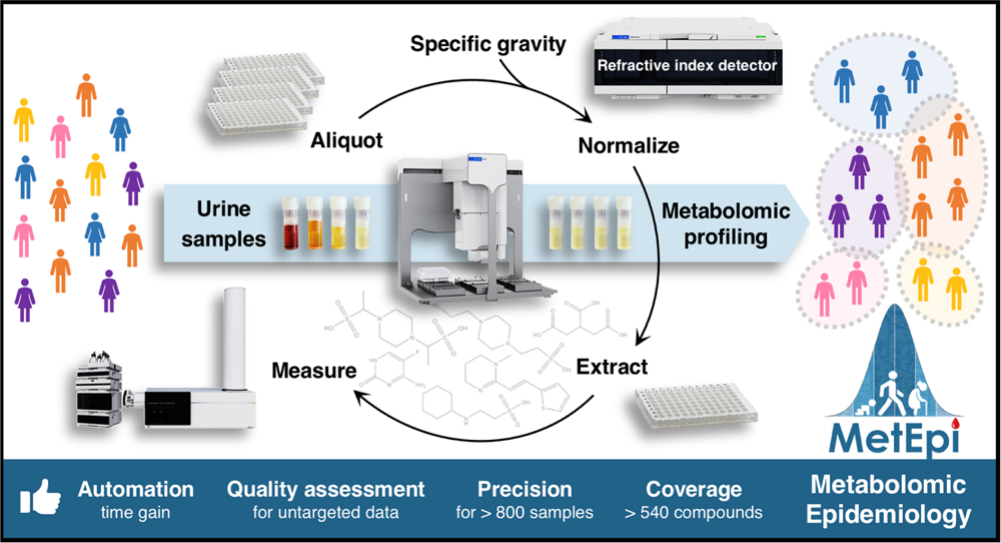

Urine is a noninvasive biofluid that is rich in polar metabolites and well suited for metabolomic epidemiology. However, because of individual variability in health and hydration status, the physiological concentration of urine can differ >15-fold, which can pose major challenges in untargeted liquid chromatography–mass spectrometry (LC–MS) metabolomics. Although numerous urine normalization methods have been implemented (e.g., creatinine, specific gravity—SG), most are manual and, therefore, not practical for population-based studies. To address this issue, we developed a method to measure SG in 96-well-plates using a refractive index detector (RID), which exhibited accuracy within 85–115% and <3.4% precision. Bland–Altman statistics showed a mean deviation of −0.0001 SG units (limits of agreement: −0.0014 to 0.0011) relative to a hand-held refractometer. Using this RID-based SG normalization, we developed an automated LC–MS workflow for untargeted urinary metabolomics in a 96-well-plate format. The workflow uses positive and negative ionization HILIC chromatography and acquires mass spectra in data-independent acquisition (DIA) mode at three collision energies. Five technical internal standards (tISs) were used to monitor data quality in each method, all of which demonstrated raw coefficients of variation (CVs) < 10% in the quality controls (QCs) and < 20% in the samples for a small cohort (*n* = 87 urine samples, *n* = 22 QCs). Application in a large cohort (*n* = 842 urine samples, *n* = 248 QCs) demonstrated CV_QC_ < 5% and CV_samples_ < 16% for 4/5 tISs after signal drift correction by cubic spline regression. The workflow identified >540 urinary metabolites including endogenous and exogenous compounds. This platform is suitable for performing urinary untargeted metabolomic epidemiology and will be useful for applications in population-based molecular phenotyping.

## Introduction

The use of metabolomics is increasing in clinical research and metabolomics data have become an essential component of molecular phenotyping.^1^ In conjunction with these developments, the discipline of metabolomic epidemiology has been created, which involves the systematic use of epidemiological methods and principles to study population-based variations in the human metabolome as it associates with health-related outcomes and exposures.^2^ These efforts regularly involve the need to analyze large-scale studies of thousands of individuals, which can be a significant obstacle for many untargeted analytical methods. While progress has been made using standardized targeted metabolomics platforms,^3−5^ there are few methods for large-scale untargeted mass spectrometry-based approaches. In order to meet the demands of this field in terms of experimental throughput and data quality, there is a need to develop untargeted metabolomics methods that have high precision and can be fully automated.

Because of its ease of collection, urine is a particularly well-suited biofluid for metabolomic epidemiology and population-based molecular phenotyping. The urinary chemical composition represents an integrated snapshot of the entire organism, providing insight into systemic metabolism. However, urine poses a number of challenges for untargeted liquid chromatography–mass spectrometry (LC–MS)-based metabolomics, including the large amount of salts (inorganic salts, urea and creatinine collectively represent ∼84% of the total urine solutes^6^), wide variation according to the hydration status of the individual (urinary specific gravity, [SG], varies >15-fold^7^), and large dynamic range of metabolites (>10 orders of magnitude^8^), which can collectively result in retention time (RT) shifts and matrix effects.^9−11^ The dilution and normalization of urine samples to a uniform concentration prior to untargeted LC–MS measurements is crucial to achieve high data quality because postacquisition normalization methods are unable to fully compensate for concentration-related matrix effects.^9,12−14^ It is, therefore, a common practice to normalize the urinary concentration using a variety of methods including SG,^15^ creatinine,^16^ or osmolality.^14,17^ SG is the preferred urinary normalization method employed by the World Anti-Doping Agency (WADA)^18^ because of its ease of measurement and reduced confounding effects. SG measurements are currently performed by refractometry, while true SG measurements by gravimetry are comparatively complicated and have become obsolete.^19,20^ The refractometry SG is an indirect measurement of SG, where the refractive index (RI) observed in urine is converted to a SG value using experimental conversion tables.^20^ In practice, SG is generally measured using a hand-held refractometer, which is time-consuming and not amenable to automation.

To address these needs and challenges, we report the development of an automated untargeted metabolomics workflow for urine. In particular, a differential RI method was developed to automate the SG measurement and all sample preparation steps are performed using a liquid handling system and 96-well plates. Samples are then analyzed using a combination of positive and negative ionization HILIC chromatography to provide wide metabolic coverage of urinary metabolites.^21^ Taken together, this combined workflow presents a fully automated method for urinary untargeted metabolomics that offers the throughput and precision necessary for applications in molecular phenotyping and metabolomic epidemiology.

## Experimental Section

### Chemicals, Solvents, and Urine Samples

The complete list of solvents and chemicals, including standards used to create the spectral libraries, is provided in Table S1. LC–MS grade solvents were used throughout this work. Spectral libraries were acquired in data-dependent (DDA) and data-independent (DIA) acquisition modes.^22^ Five technical internal standards (tIS; Table S2) were selected for monitoring the system performance.

The method was characterized using fasting urine samples (*n* = 87) from subjects from the LUNg obstruction in Adulthood of PREmaturely born (LUNAPRE) study (ClinicalTrials.gov NCT02923648).^23^ Stability assessments were performed using pooled urine from all individuals (hereafter referred to as pooled study quality control sample, SQC) as well as internal laboratory reference urine from healthy volunteers (pooled laboratory reference quality control, RQC). Proof-of-concept studies for large-scale application were performed using urine samples from a rhinovirus challenge study (*n* = 842; Netherlands Trial Register NTR5426/NL5317).^24^

### Sample Aliquoting

Urine samples were thawed overnight at 4 °C, vortexed, and 1.5 mL was transferred to each well of a 2 mL 96-deepwell plate placed on a metal block on ice (instrumentation and materials are listed in Table S3). An additional aliquot of 1.5 mL of each sample was pooled to create the SQC sample, which was then transferred to the dedicated wells of the deepwell plate (Figure S1). To avoid multiple freeze–thaw cycles, the deepwell plate content was directly aliquoted in 15 aliquots of 120 μL into 0.2 mL 96-well plates using a Bravo automated liquid-handling platform (Agilent Technologies, Inc.) equipped with a cooling unit set at 4 °C using 250 μL tips. Aliquot plates were sealed with peel seals on a thermal microplate sealer for 2 s at 170 °C and stored at −80 °C until use.

For all subsequent sample-processing steps, one aliquot plate was placed on a metal insert for 1 h at 4 °C for uniform thawing. The plate was then shaken for 10 min at 1600 rpm and 4 °C in a thermomixer, centrifuged at 4390*g* in a plate centrifuge for 40 min at 4 °C, and 105 μL of the supernatant was pipetted using the liquid handler to a new 0.2 mL plate.

### Specific Gravity Measurement

Manual measurements of SG were performed using an UG-D hand-held refractometer (Atago, Inc.). Samples were equilibrated to room temperature prior to SG measurements. The refractometer was calibrated with high-purity water, wiped with a lint-free tissue after each measurement, and recalibrated after every 20 samples.

Automated SG measurements were performed using an Agilent 1260 differential refractive index detector (RID) coupled to an Agilent 1290 multisampler and an Agilent 1260 quaternary pump with high-purity water as the single mobile phase. In contrast to the static RI cell of the hand-held refractometer, the differential RI is a single flow-through cell divided into two compartments: the reference compartment (filled with the mobile phase and is static over the time of measurement) and the sample compartment (receives urine samples delivered in the mobile phase). As the urine passes into the sample cell, the light beam is diffracted, producing a differential with the reference cell, which is translated into nano RI units (nRIU). The mobile phase flow rate was set at 2 mL/min, the urine injection volume was 0.6 μL, and the optical unit temperature was 35 °C. The total run time was 0.09 min per sample (<0.5 min total injection run time) and the acquisition rate was 37 Hz. OpenLab 2.4 software was used to control the system and integrate the RID signal nRIU peak areas. For the conversion of nRIU peak area values into SG, a 10-point calibration curve was prepared in NaCl by a serial dilution of a 2 M stock solution in water with corresponding SGs of 1.002–1.057 measured with the UG-D refractometer. A commercial 1 M NaCl solution was used to prepare the QC samples for the calibration, with 1 M as the highest QC, and further diluted 2-, 10-, and 20-fold with water for the middle, low, and LLOQ QCs. These correspond to SG values of 1.030, 1.015, 1.003, and 1.002 on the UG-D refractometer, respectively. Sample preparation and measurement parameters are detailed in Table S4. A linear calibration curve with 1/*x*^2^ weighting was applied. Intra and interday accuracy and precision were assessed following the FDA guidelines.^25^ All estimates were calculated with SG values-1 to avoid underestimation of accuracy and precision values because of the narrow range of values compared to the blank value (the SG of water is 1.000). Manual versus automated SG measurements were compared using Bland–Altman plots in *R*.^26^

### Sample Preparation

Urine SG normalization was performed on the liquid handler cooling unit set at 4 °C and equipped with a metal insert. High-purity water was used to dilute urine to a common SG value (1.002) in 0.45 mL 96-well plates. SG normalization was performed using the equation adapted from Levine and Fahys^27^ volume_urine_ = total_volume_*(SG_target_ – 1)/(SG_sample_ – 1), where the total volume is 340 μL and the target SG value is 1.002 (lowest normal physiological value^7^). To avoid evaporation during the 2.7 h of the SG normalization process, aliquot plates and SG-normalized urine plates were covered with slit seals.

Depending on the LC–MS method, SG-normalized urine was diluted to 100 μL with acetonitrile containing tIS in 0.2 mL 96-well plates using the liquid handler (1:9 v/v for positive ionization or 3:7 v/v for negative ionization, see Table S2 for tIS concentrations), sealed with peel seals and incubated at 4 °C for 2 h. Plates were centrifuged at 4390*g* and 4 °C for 40 min, followed by transfer of 50 μL of the supernatant to new 0.2 mL plates using the liquid handler, and then sealed with pierce seals for 3.5 s at 180 °C for direct use in the LC–MS multisampler.

### LC–MS Measurements

LC–MS settings for the measurement of urine samples are detailed in Table S5. Briefly, samples were measured on an Agilent 1290 Infinity II ultrahigh-performance liquid chromatography system coupled to a 6550 iFunnel quadrupole-time-of-flight mass spectrometer equipped with a dual AJS electrospray ionization source tuned for the 50–750 *m*/*z* range. The positive mode chromatography (ZHP) was adapted from Naz *et al.*(28) and Chaleckis *et al.*(29) using a SeQuant ZIC-HILIC column and a gradient between (A) water containing 0.1% formic acid (pH = 2.6) and (B) acetonitrile containing 0.1% formic acid. The separation gradient included an isocratic step at 95% B for 1.5 min followed by a gradient to 40% B in 10.5 min. The negative mode chromatography (ZHN) was run on a SeQuant ZIC-pHILIC column with (A) ammonium acetate 5 mM with 0.04% ammonium hydroxide in water (pH = 9.3) and (B) pure acetonitrile as mobile phases. The gradient was set at 88–60% B from 0 to 8.5 min and the column oven was heated at 35 °C. The acquisition was performed in DIA mode using a mass range of 40–1200 *m*/*z* with three different collision energies (0, 10, and 30 eV). The ZIC-pHILIC method was developed using a previous column version equipped with PEEK frits. However, the manufacturer changed the frit material from PEEK to titanium in 2019, resulting in exceeding the column pressure limit during the wash step. The wash and re-equilibration steps have been subsequently modified to remain within the column pressure limits for the current column version as described in Table S5.

### Data Availability

The LUNAPRE study datasets have been deposited in the EMBL-EBI MetaboLights repository^30^ with the identifier MTBLS2295. Chemical standard RTs were submitted to PredRet^31^ and MS spectra to MoNA (MassBank of North America).

### Data Quality Check and Preprocessing

The data quality check and preprocessing procedure are described in the Supporting Information and Tables S6–S10. Briefly, raw files were converted to mzML using ProteoWizard^32^ and an initial quality check was performed in MZmine 2.53^33^ (Table S6) using a predefined list of metabolites (Table S7). For preprocessing of the annotated dataset, mzML files were converted to the “Analysis Base File” (ABF) format using Reifycs Abf Converter and loaded into MS-DIAL 4.20^34^ (Tables S8 and S9).^35^ The MS2 spectra were deconvoluted using MS2Dec^35^ and CorrDec.^36^ Identifications were based on in-house compound libraries containing 404 and 295 chemical standards for the ZHP and ZHN platforms, respectively (Table S1 and previous studies).^22,28^ Metabolites annotated by MS-DIAL were further curated using the following criteria: a RT shift <0.5 min, a mass shift <10 mDa, and, for spectral match, both a dot product score without weighting above 700 and at least three matching MS^2^ peaks with the reference spectra to avoid spurious high scores from too low number of peaks.^37^ Any annotations that did not fulfill one of the RT or *m*/*z* criteria, but were still determined to be accurate received an explanatory comment in the identification tables. Peak areas were exported from MS-DIAL. Using R scripts,^26^ coefficients of variation (CVs) and mean peak intensities were computed for the SQC samples, missingness (percentage of samples below 5× the blank signal for a given metabolite), skewness, interquartile range (IQR), and D-ratios (percentage ratio of the SQC to sample standard deviations^38^) were calculated in the samples for all annotated metabolites. For preprocessing the all-feature dataset, peak detection at the MS1 level was performed in MZmine (Table S10).^39^ Peak heights were exported from MZmine to be filtered in R using the following criteria: CV in the SQCs <35%, peak heights within the dynamic range of the instrument (for the system reported here: 1.5 × 10^3^ to 3.5 × 10^6^), missingness < 90%, D-ratio < 55%, IQR > 80. MS-DIAL (annotated) and MZmine (all-feature) dataset signals were corrected for measurement drift using a Matlab algorithm based upon the SQC signals.^38^

### Stability Assessment of the Urine Samples

The stability during the normalization process on the liquid handler was tested by leaving normalized urine samples on the cooling deck for 3 h (the time required to normalize a full 96-well plate) compared to 3 h on wet ice and 3 h at room temperature. The stability in the multisampler was evaluated by placing the processed (extracted with acetonitrile) sample plate in the multisampler set at 4 °C for 50 or 96 h (required time to measure 1 or 2 plates) compared to urine processed and measured immediately. We also evaluated whether short-term storage of the processed urine at −80 °C (implying a freeze–thaw cycle) was more advantageous than leaving the urine in the multisampler queue for days. The stability to 1 versus 3 freeze–thaw cycles was assessed for normalized urine samples by allowing urine samples stored at −80 °C to thaw at 4 °C for 4 h, which corresponds to 2 h of thawing and 2 h of the normalization time. Prior to the experiment, both the SQC and RQC samples experienced at least two additional freeze–thaw cycles. The storage time stability at −80 °C during 2 and 10 months was compared between unprocessed urine, normalized urine, and processed urine.

## Results and Discussion

### Workflow Description

**Figure 1:**
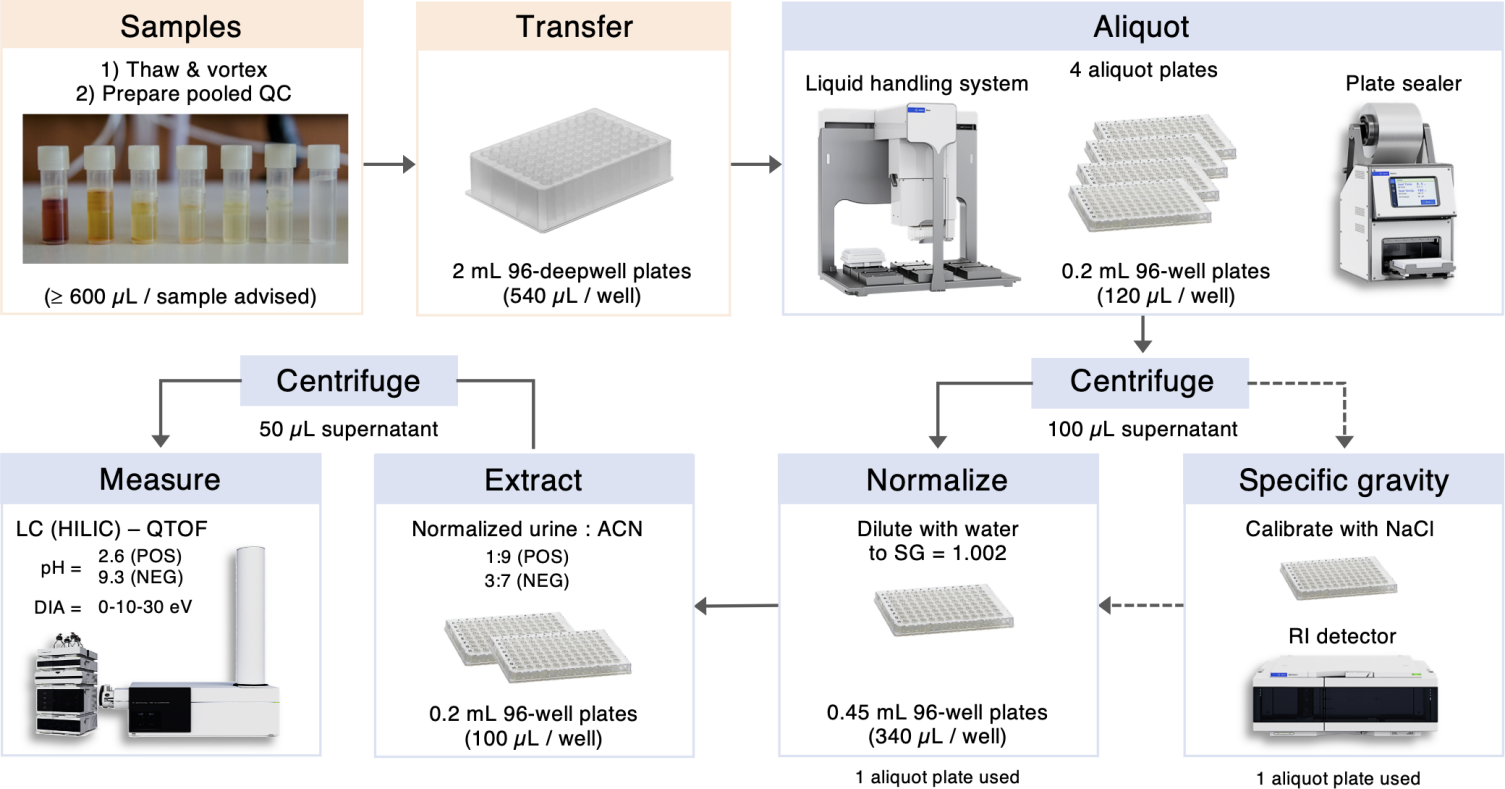
Urinary untargeted metabolomics workflow. Manual steps are framed in orange and automated steps are shown in blue. QC = quality control; RI = refractive index; SG = specific gravity; POS = positive ionization; NEG = negative ionization; and DIA = data independent acquisition.

We present here an untargeted LC–MS-based metabolomics workflow for the automated processing of urine samples. The use of the 96-well plate format enables the sample preparation to be primarily performed using an automated liquid handing platform (Figure 1). The workflow incorporates batch structures that can be used in both small and large studies, and contains multiple QC samples to monitor instrument performance across the analytical runs as well as for eventual signal correction (Figure S1).^38,40,41^ Because of the large number of performance parameters and associated metabolites to evaluate, this workflow was developed and characterized using a small study of 87 urine samples.^23^ As proof-of-concept for application in larger cohorts, the workflow was then tested with a cohort of 842 urine samples.^24^

A major advantage of working with urine is that it is available in large quantities and relatively easy to obtain. As part of the workflow proposed herein, we recommend a starting volume of 600 μL of urine (Figure 1), of which 540 μL are transferred to 2 mL 96-well plates and 40 μL are used to create the SQC sample, which is aliquoted into 20 wells per plate (Figure S1). To reduce freeze–thaw cycles, the 2 mL urine plates are then aliquoted into four plates (120 μL/well), of which one is used for SG measurements, two are dedicated to metabolomics measurements, and one serves as an eventual back up. The urine normalization protocol results in a normalized urine volume of 340 μL per well (see Supporting Information for a detailed description of the protocol). Because the extraction protocol only uses a maximum of 30 μL of the normalized urine per platform, the remaining urine can be stored as needed.

The method was developed with an analytical batch based upon a 96-well plate containing 70 samples, 20 SQCs, 2 RQCs, and 4 blanks (Figure S1). Given that the small cohort for method development was just over the maximal allowance for one plate, samples were equally divided between two plates and measured as a single batch. The injection sequence followed recommendations from Broadhurst et al*.*^38^ with samples interspaced with SQCs after each 5th sample (Figure S1 and Table S11).

The gains in automating the sample preparation workflow can be described in terms of operator time (Table S12). Overall, the automation resulted in an almost 7-fold gain in person-time per plate (from 6.9 to 1 h), and decreased operator fatigue and related errors. For application in large-scale cohorts, for example, processing 1050 samples in 15 batches, the total gain in time for the automated protocol is 88.6 h, equating to ∼11 days of full-time work. Further automation of the workflow can be achieved by using sample tubes for urine collection that can be employed directly in the sample handler, eliminating the need for manual manipulation of the sample tube (e.g., Thermo Matrix rack system tubes).

### Urine Normalization

Normalization of the urinary solute concentration is a crucial step because of physiological fluctuations in the matrix composition.^9^ Multiple normalization strategies have been proposed including urinary SG,^15^ creatinine,^16^ and osmolality.^17^ Creatinine is widely used, but is susceptible to interindividual characteristics including age, sex, diet, and muscle cachexia.^42,43^ The WADA has adopted SG because of its general applicability.^18^ In addition, given the time-consuming nature of normalizing the urine samples to a common dilution factor prior to LC–MS analysis, some efforts have applied either normalization of the MS signals postacquisition^17^ or injection of variable urine amounts.^12^ The main issue of postacquisition normalization is that there are significant matrix effects that stem from the high variability in the concentration and composition of urine. This can lead to ion suppression or enhancement as well as solid phase binding competition, which are all analyte-specific.^9,11^ In contrast, targeted methods that employ internal standards generally provide good results with postacquisition normalization.^44^ However, it is not possible to correct for these analyte-specific matrix effects in untargeted metabolomics. To demonstrate these effects, we measured a small cohort (*n* = 87 samples) with no-normalization as well as pre and postacquisition urinary SG normalization (Figure S2).

The WADA protocol for measurement of urinary SG uses a hand-held refractometer, which converts the RI of urine to the corresponding SG.^45,46^ We tested the reproducibility of refractometry SG readings between laboratories (in Japan and Sweden), as well as their stability after five freeze–thaw cycles (Figure S3). The reproducibility of SG readings between laboratories is good, with a mean bias of 0.0001 (limit of agreement, LOA: −0.0009 to 0.0012). The effects of five freeze–thaw cycles on urine SG readings translated to a small bias of 0.0008 (LOA: −0.0002 to 0.0019).

**Figure 2:**
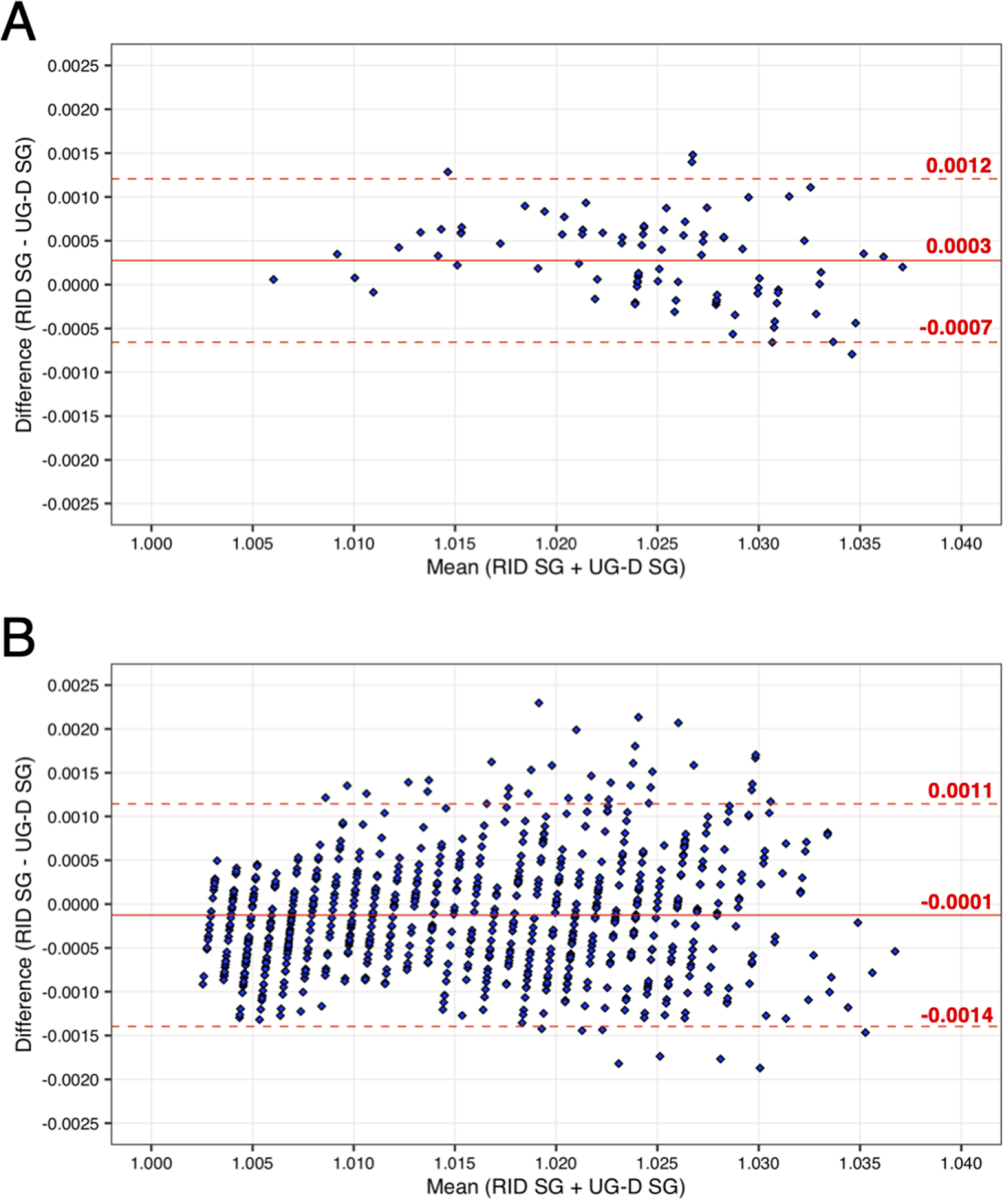
Bland–Altman plots of urine SG measured with a hand-held refractometer (model UG-D) vs RID. (A) The small cohort (*n* = 87). (B) The large cohort (*n* = 842). Each sample is represented by a blue diamond. Mean deviation is shown as a solid red line with the 95% confidence intervals (limits of agreement) as red-dotted lines.

The measurement of SG using a hand-held refractometer is time-consuming for large sample numbers and is a significant bottleneck for automating metabolomics. We, therefore, developed an automated 96-well plate format method to measure RI using an multisampler connected to a RID. The RID measurements are converted to SG using a calibration curve consisting of NaCl, with SG measured with a refractometer. This method was validated with the UG-D refractometer model (available only in Japan) and the UG-α model (corresponding international version) that are based on a slightly different SG conversion.^45,46^ Intra and interday accuracy and precision were within the recommended ±15% thresholds (Table S13), except for the UG-D intraday NaCl QC low (117%), which can be attributed to the 3-digit SG reading precision of this model. Comparison of SG measurements obtained from RID versus the refractometers showed a mean bias of 0.0003 for the UG-D (LOA = −0.0007 to 0.0012; Figure 2A) and a mean bias of −0.00003 for the UG-alpha (LOA = −0.0009 to 0.0008; Figure S4). The RID measurements show a good agreement to both hand-held refractometer values, demonstrating the utility of the RID method. In addition, the automation of the SG measurements can increase biosafety because of the decreased transfer of the urine by hand.

### Analytical Methods and Data Preprocessing

Given that urine is the main route for the elimination of water-soluble waste products, we propose here a combination of two analytical methods using HILIC chromatography. The HILIC method at pH = 2.6 in positive ionization (ZHP) was adapted from previously published work^28,29^ and a new method was developed for negative ionization at pH = 9.3 using a ZIC-pHILIC column (ZHN).

Features were annotated in MS-DIAL using accurate mass (AM) and RT match, as well as spectral match (MS/MS) to our in-house libraries, and MS-FINDER 3.42^47^ for the identification of unknown compounds. For the ZHP dataset, of the raw 10,363 features, 406 features were annotated (including fragments and adducts), their integration checked and their CVs across SQCs and D-ratios calculated, as well as intensity plots across the injection sequence. After filtering for single-species and CV values, the final ZHP annotated dataset was comprised of 295 metabolites (Table S14), of which 126 were AM, RT, and MS/MS matched, 73 were AM and RT matched, and 96 were AM matched with MS-FINDER. For the ZHN dataset, 465 out of 9332 features were annotated, resulting after filtering in 358 single metabolites (47 AMRT-MS/MS, 87 AMRT, and 224 AM; Table S15). Median absolute AM and RT differences were 0.3 mDa and 0.2 min for the ZHP method and 0.3 mDa and 0.03 min for the ZHN method.

Because the annotated dataset does not include unknown peaks, we processed an additional dataset at the feature level (a feature is defined as a pair of *m*/*z* and its corresponding RT). The all-feature dataset as exported from MZmine underwent several additional filtering steps in R, including: CV_QC_ < 35% (filtering out signals displaying low precision), D-ratio < 55% (removing signals with low biological to SQC technical variability ratio), and IQR > 0 (to eliminate features with low variability). In the ZHP untargeted dataset of 10,797 features, 632 (6%), 1320 (12%), and 33 (0.3%) entries were identified as falling outside the respective CV, D-ratio, and IQR thresholds, while, for the 8,966 ZHN features, 503 (6%), 925 (10%), and 24 (0.2%) were outside CV, D-ratio, and IQR thresholds.

### Metabolomic Coverage of Urine Components

**Figure 3:**
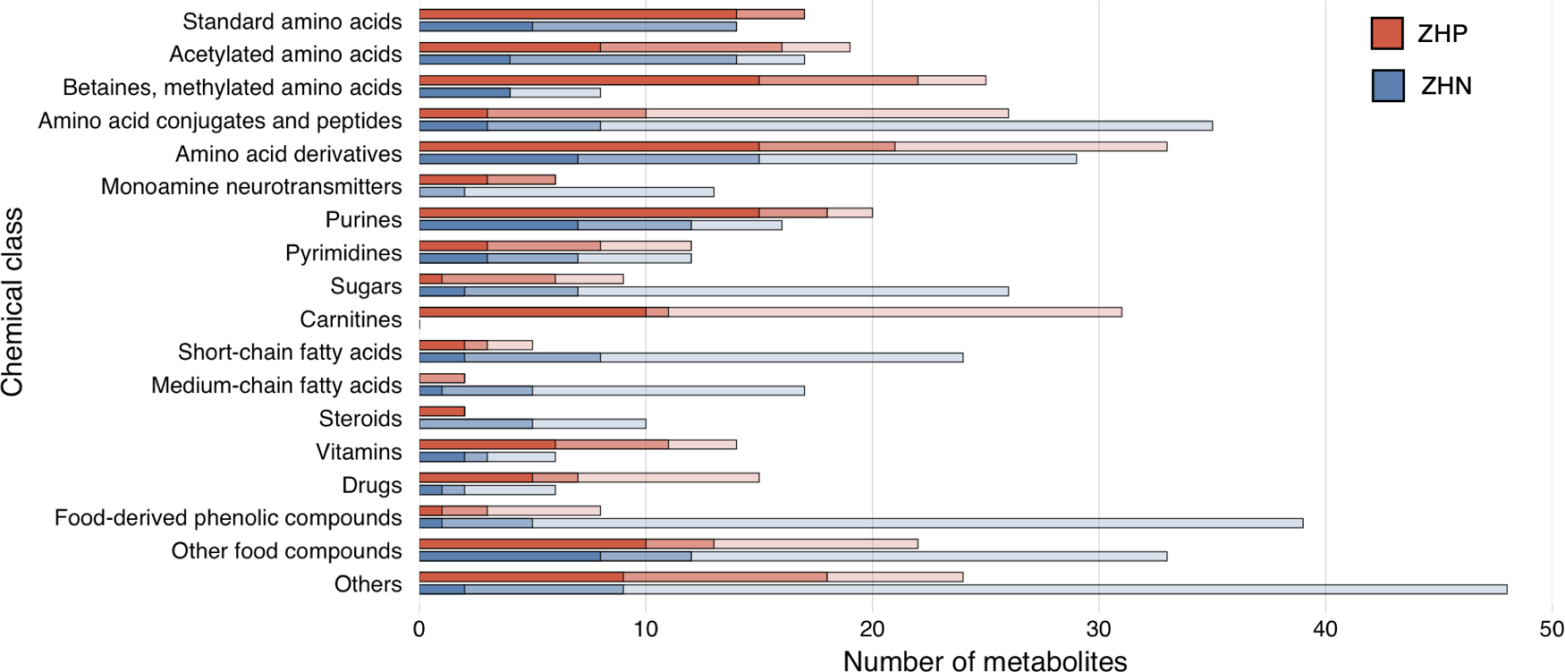
Metabolic coverage and annotation confidence levels. Annotated compounds are provided based upon chemical class. Shades of red indicate metabolites from the ZHP platform. Shades of blue indicate metabolites from the ZHN platform. The darkest shades indicate compounds that matched to the in-house library with AM, RT, and MS/MS spectrum; the mid-level shades indicate compounds that matched to the in-house library, but lack a spectral match; the lightest shades indicate compounds that are identified with AM from external databases (most of the time also using in silico spectral match). ZHP = ZIC-HILIC positive ionization and ZHN = ZIC-pHILIC negative ionization.

To offer broader coverage of urine components using untargeted LC–MS methods, we chose two complementary HILIC methods in positive and negative ionization. The methods overlap for 109 compounds, while providing unique coverage of 186 and 249 compounds for ZHP and ZHN, respectively. Taken together, 544 unique metabolites were detected covering major chemical classes, including amino acids, nucleobases, lipids, vitamins, and exogenous compounds (e.g*.*, diet-derived, medicines) (Figure 3). Both methods perform equally well for the majority of amino acids and their derivatives. However, the combination of two chromatographies and polarities enables the coverage of specific compound classes. The ZHP method offers an extensive coverage of acylcarnitines and betaines, while the ZHN method outperforms for the detection of lipids (SCFA, MCFA, and steroids) and sugars as well as microbiota- and diet-derived compounds. The performance in the latter category is mostly because of phase II conjugated compounds, such as sulfated, glucuronides and other glycosylated species that account for >25% of the total annotations. These results stress the importance of phase II metabolic products in urine for characterizing dietary patterns. There are few available standards for this class of compounds, and instead enzymatic treatment combined with MS methods followed by custom synthesis have been proposed as a strategy to rapidly identify these metabolites.^48^ Accordingly, the untargeted ZHN platform developed for the current study is useful for providing a snapshot of these metabolic processes as well as promoting structure characterization and long-term identification efforts.

We evaluated our coverage of urinary metabolites by comparing our annotations to the current Urine HMDB repository (4364 entries as of 2020-09-10).^8^ We observed an overlap of 286 compounds, while our methods include 258 compounds not yet reported in Urine HMDB. As this repository provides the concentration data, we also compared our annotation list with a subset of 1199 compounds with available urine concentrations in healthy adults. Of the 211 compounds with urine concentrations and overlapping with our annotations, 89% had mean concentrations >0.1 μmol/mmol creatinine, which could be taken as an overall estimation of our platform sensitivity.

We also assessed our coverage compared to two other recent metabolomics platforms: one offering a coverage of 142 urinary compounds, of which half can be quantified,^49^ and another targeting exposome research with a wide coverage of endogenous and exogenous compounds quantifying 690 compounds for a total of 1022 annotations.^50^ Of the first study, only 60 compounds overlap with our annotations, because of the strong focus on urinary lipids that are for most only present in low abundance and for which targeted MS/MS is necessary. When comparing our coverage with the exposome platform, we observed an overlap of 222 compounds, with 318 uniquely reported by our methods. Here again, the use of a targeted MS/MS approach, which is well suited to detect low abundant compounds, can explain the relatively small annotation overlap. However, by definition, targeted approaches can only focus on already known compounds, while our untargeted methods can perform de novo annotations at the MS^2^ level. Given that our general sensitivity threshold is ∼0.1 μmol/mmol creatinine, it is likely that the 258 compounds detected by our method, but not yet reported in urine are in fact present in relatively high abundance. These findings support the use of untargeted metabolomics to map unknown components in urine.

### Precision of the Workflow

**Figure 4:**
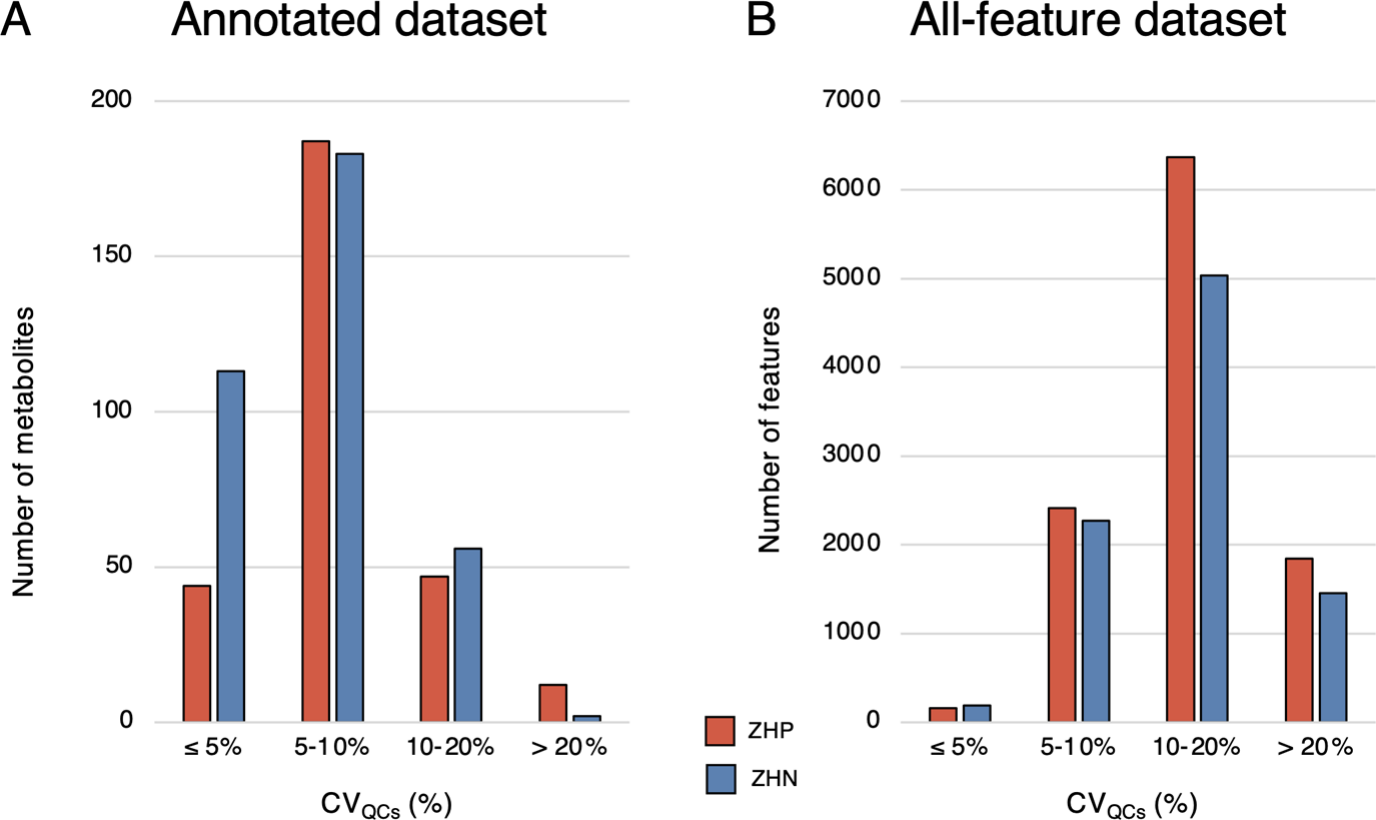
Bar plot of metabolites or feature CV in each of the platforms. (A) Annotated metabolite datasets with the ZHP platform represented by blue bars (*n* = 295 metabolites) and the ZHN platform by red bars (*n* = 358); (B) all-feature datasets (*n* = 10,795 features in ZHP and *n* = 8,961 in ZHN). Data are from the 22 pooled SQC samples from the small cohort. ZHP = ZIC-HILIC positive ionization and ZHN = ZIC-pHILIC negative ionization.

In contrast to targeted methods, untargeted metabolomics aims to measure hundreds to thousands of potentially unknown metabolites. It is not possible to assess the data quality using the standard targeted metrics of accuracy and precision for each feature. To address this issue, we implemented tIS to monitor analytical performance. These selected standards are exogenous compounds that are not generally found in biofluids, are readily available, and are affordable (Table S1 and Figure S5).^51^ In automated liquid-handling systems, the extraction solvent containing the tIS is dispensed from a reservoir containing excess volume, justifying the need for affordable standards. Moreover, isotopically labeled metabolites might interfere with deconvolution of the untargeted data, complicating spectral information. The use of tIS enables evaluation of the LC–MS system prior to initiating an analytical run and provides a means to monitor the precision of the measurements within and between analytical batches across the entire LC–MS workflow. For example, shifts in RT, injection volume inconsistencies, or mass spectrometer ionization issues can rapidly be identified and the potential impact on the measurements assessed. Based upon our experience with both platforms, we set acceptance criteria for the analytical run to be CVs in the SQCs <10% and CVs across samples <20%. When measuring the small cohort, we observed good compliance with the acceptance criteria with tISs displaying CVs <5 and <8% in the SQCs and samples, respectively, for the ZHP method, while for the ZHN method, the CVs were <11 and <20% in the SQCs and samples, respectively (Table 1 and Figure S5). For the 290 annotated single species metabolites measured in the ZHP platform prior to drift correction, 231 have CV ≤ 10%, with only 12 metabolites above the 20% threshold set by Klåvus *et al.* (Figure 4).^52^ Similarly, out of 354 annotated metabolites in the ZHN platform, 296 display CV ≤ 10% and only 2 have CV >20%. In the raw all-feature ZHP and ZHN datasets also, >80% of the features display CV_QCs_ ≤ 20%.

**Table 1:** Performance of Technical Internal Standards (tISs) at the Large Cohort Scale^a^

ZHP	raw data small cohort	raw data large cohort		pre QC-correction	post QC-correction
standard	CV_QC_/CV_sample_	mean CV_QC_ (min–max)	mean CV_sample_ (min–max)	CV_QC_/CV_sample_	CV_QC_/CV_sample_
pyrantel	3.0/2.5	2.7 (1.0–4.8)	3.7 (1.5–6.8)	23.0/23.0	2.3/3.7
CHES	3.7/6.2	3.6 (1.4–6.9)	6.8 (4.1–16.0)	22.0/23.0	4.5/9.1
fluorocytosine	3.8/7.0	8.5 (2.3–19.0)	16.0 (5.5–31.0)	40.0/40.0	19.0/19.0
PIPES	4.0/3.5	4.4 (1.7–9.2)	6.7 (3.1–13.0)	20.0/19.0	4.3/8.8
HEPES	4.4/3.1	4.5 (1.5–9.2)	6.2 (1.9–15.0)	20.0/21.0	3.8/7.0

ZHN	raw data small cohort	raw data large cohort		pre QC-correction	post QC-correction
standard	CV_QC_/CV_sample_	mean CV_QC_ (min–max)	mean CV_sample_ (min–max)	CV_QC_/CV_sample_	CV_QC_/CV_sample_
fluorocinnamic acid	10.0/20.0	5.9 (2.8–12.0)	12.5 (5.9–18.0)	15.0/23.0	2.5/16.0
CHES	4.1/6.3	4.4 (2.7–7.0)	6.9 (4.9–11.0)	12.0/16.0	2.8/8.9
HEPES	7.5/8.2	5.3 (1.5–7.9)	9.4 (6.7–12)	19.0/20.0	3.6/11.0
PIPES	5.7/10.0	4.7 (2.4–7.4)	10.0 (6.2–16.0)	25.0/27.0	3.2/12.0
tricarballylic acid	5.6/18.0	12.4 (6.8–18.0)	36.5 (14.0–66.0)	32.0/54.0	10.0/44.0

a CVs of the peak area across 24 plates (*n* = 842 samples, *n* = 248 SQCs). Raw data are CV values per plate, while pre- and post-QC correction CVs are calculated at the whole cohort scale before and after applying the QC correction algorithm.^38^ SQCs = pooled study QCs; ZHP = ZIC-HILIC positive ionization mode; and ZHN = ZIC-pHILIC negative ionization mode.

### Metabolite Stability within the Workflow

The stability of the metabolites was assessed for the entire sample preparation protocol and LC–MS measurement. In the transfer from the manual to automated workflow, the samples could no longer be processed on ice. We accordingly complemented the liquid handling system with a cooling unit and metal inserts for the 96-well plates. This provided cooling down to 6 °C in plates on metal blocks and <10 °C for the reservoirs. All manipulations by the liquid handler can be performed within 10 min, except for the urine normalization, which requires a maximum of 3 h for a full plate. We, therefore, assessed the stability of urine samples on the cooling deck of the liquid handler for 3 h compared to incubation on wet ice or at room temperature for raw and normalized SQC and RQC urine samples. Of the >200 metabolites that could be reliably observed in these two urines, <6% were affected by any of the conditions and displayed a >20% change (Table S16 and Figures S6 and S7).

Another potential issue affecting metabolite stability was the time spent in the multisampler (set at 4 °C). In our setup, the temperature in the bottom compartment is < 5 °C, while the upper compartment generally does not achieve temperatures <8 °C. Less than 6% of the metabolites monitored showed a change in intensity after 50 or 96 h at 5 °C. However, a short-term storage of extracted urine at −80 °C is not advised for the ZHP method; this additional freeze–thaw led to a decrease of >20% in intensity for 40% of the metabolites in the RQC (12% of the metabolites changed in the SQC), while it had virtually no effect upon ZHN metabolites (<1% affected). During the sample processing, there is the option to freeze normalized urine and/or extracted urine for later use. We, therefore, investigated the stability of metabolites stored in normalized or extracted urine and observed minimal differences (<11% of the metabolites were affected) between unprocessed, normalized, and extracted urine when stored for 2 weeks, and up to 10 months. Finally, we also demonstrated the stability of normalized urine after several freeze–thaw cycles (>85% were stable) for the ZHP method, while metabolites from the ZHN method were more sensitive, especially for the SQC urine (while a single freeze–thaw cycle had a minimal effect, >50% were affected following three freeze–thaw cycles) (Table S16, Figures S6 and S7).

### Performance in a Large Cohort

We tested the applicability of our workflow for a large cohort (*n* = 842 samples). We first evaluated SG measurements performed with the developed RID method compared to measurements with the UG-D refractometer. The method showed excellent agreement between the refractometer and the RID, with a mean deviation of −0.0001 and LOA = −0.0014 to 0.0011 (Figure 2B). These findings demonstrate the suitability of the RID SG measurement method for large sets of urine samples. In addition, this RID method might be of use for normalizing other less common biofluids. Initial trials with saliva were encouraging (data not shown).

In the case of large study sizes, on the scale of >1000 samples, analytical measurements can last several weeks and the probability of encountering technical issues significantly increases. As proof-of-concept for the application of the present workflow for large cohorts, we provide in Table 1 CVs for 842 urine samples measured in 24 batches (1090 urine samples in total, including 248 SQCs).

For the ZHP platform, 4 tISs (pyrantel, PIPES, CHES and HEPES) met the acceptance criteria of CV_QCs_ < 10% and CV_samples_ < 20% across all 24 batches. Fluorocytosine did not meet the criteria in eight plates for the CV_QCs_ and the CV_samples_. This tIS elutes in a busy RT window and is injected at >5 times lower concentration than the other tIS because of a strong ionization. For the ZHN platform, 4 tISs (fluorocinnamic acid, CHES, PIPES, and HEPES) also displayed CVs generally below set thresholds (fluorocinnamic acid exceeded CV_QC_ threshold values in one plate). The fifth tIS (tricarballylic acid) evidenced a higher variability, especially in CV_samples_. This tIS elutes at the far end of the gradient, where the percentage of water in the mobile phase is high and desolvation more difficult. Also, it elutes closely to two abundant metabolites (citric acid and ascorbic acid sulfate) that interfere with the signal. While fluorocytosine and tricarballylic acid demonstrated high variability, they are still of utility because they demonstrate potential performance issues at those RT in the chromatograms and can be used as anchors for RT correction algorithms. The remaining tISs display a high precision at the large cohort scale. In the case when a tIS exceeds threshold values, a more thorough investigation is performed. For example, in one plate, CHES showed a slightly higher CV. By plotting the intensities across injections, we observed two samples with markedly lower CHES abundance (Figure S8). The 3D plots revealed a highly abundant compound that eluted close to CHES, which was identified as the antibiotic trimethoprim and was suppressing the CHES signal. Therefore, tIS performance should be carefully assessed depending on the context of the urinary cohort, especially when the urine sample strongly deviates from the usual composition. The performance of these tISs also demonstrates the analytical challenge of untargeted metabolomics.

After drift correction, we report CV_QCs_ < 5% and CV_samples_ < 16% for 4/5 tIS for the entire dataset of 1090 chromatograms in both ZHP and ZHN. In a recent targeted metabolomics study with 690 reference standards,^50^ the authors report a mean intraday precision of 6.9% (range: 0.1–16.1%) as calculated in five pooled QCs, which are close to our CV_QC_ values at the single batch level, and mean interday of 10.9% (1.5–19.6%), markedly higher than our mean postcorrection tIS CV_QCs_. In another targeted platform used on 1800 samples and measuring 142 urinary metabolites,^49^ intraday CVs ranged from 0.23 to 8.34% and interday CVs from 0.77 to 12.8% in triplicate pooled QCs, which are similar to our reported tIS CVs. Also, considering that >75% of our raw peak intensities show CV_QCs_ ≤ 10% in both annotated datasets and >82% of the raw features have CV_QCs_ ≤ 20%, our untargeted workflow provides a precision level similar to recent targeted metabolomics platforms.

## Conclusions

The workflow presented here offers an automated platform for urinary profiling in metabolomic epidemiology. The adaptation to a 96-well plate format enabled automation while increasing the potential for high throughput. The implementation of a novel RID method for high-throughput measurement of urinary SG offered significant savings in operator time, while also developing a method that can be of utility for rapidly measuring SG in nonmetabolomics studies. The untargeted methods cover a wide range of known urinary metabolites, while enabling the discovery of novel compounds. We also provide metrics for the quality assessment of analytical runs. There are, however, limitations in the method including the variability in metabolite RT inherent to HILIC columns as well as time-consuming sample acquisition and data processing. For example, even with the automated sample preparation, the estimated acquisition time for a single 96-well plate is ∼40 h per polarity. This rate of acquisition would require ∼3 weeks of instrument time per polarity for the 842-sample study reported herein. Accordingly, parallel instruments and/or dual LC column systems will be necessary in order to achieve truly high-throughput acquisition. In addition, in spite of the excellent performance of 4/5 tISs in a large cohort, there is still variability associated with the performance of some metabolites in both ZHP and ZHN, highlighting that the data from untargeted metabolomics require confirmation with targeted methods using the appropriate internal standards. Limitations aside, the method offers a number of advancements that will be of utility in increasing the sample throughput as well as the quality of the acquired data. Taken together, the developed platform offers the potential for automated urinary metabolomic epidemiology analyses with a high precision. This will be useful for performing large-scale studies and molecular phenotyping efforts as part of a personalized medicine strategy.

## Supporting Information Available

Detailed description of urine normalization, data quality check, and data preprocessing; extended discussion of sample preparation and analytical methods; analytical batch layout and sample organization on the 96-well plate (Figure S1); effects of urine normalization upon the multivariate data structure (Figure S2); Bland-Altman plots of inter-laboratory refractometry specific gravity readings and freeze-thaw cycle consistency (Figure S3); Bland-Altman plots of refractometry-based specific gravity readings with RID-based readings (Figure S4); overlaid chromatograms of the technical internal standards (tIS) (Figure S5); stability of the ZHP platform annotated metabolites across the reported workflow (Figure S6); stability of the ZHN platform-annotated metabolites across the reported workflow (Figure S7); effect of the coeluting antibiotic trimethoprim on the intensities of the technical internal standard (tIS) CHES (Figure S8); and creatinine molecular ion (A) and reporter ion (B, dimer) intensities (Figure S9) (PDF)
Lists of chemicals and instrumentation, LC–MS and RID settings, data preprocessing parameters and supporting files, list of identified metabolites, stability data, and workflow timings (XLSX)
