# Supplementary material for: High-Precision Automated Workflow for Urinary Untargeted Metabolomic Epidemiology
Source: Anal Chem. 2021 Mar 19;93(12):5248–58. doi: 10.1021/acs.analchem.1c00203 (PMC8041248; doi:10.1021/acs.analchem.1c00203)
Supplement: Supplementary file 1 — ac1c00203_si_001.pdf [file ac1c00203_si_001.pdf]

Supplementary Information

High-precision automated workflow for urinary untargeted metabolomic epidemiology

Isabel Meister[1,2], Pei Zhang[1,2,†], Anirban Sinha[3,4,5], C. Magnus Sköld[6,7], Åsa M. Wheelock[6,7], Takashi Izumi[1,8], Romanas Chaleckis[1,2,*,‡], Craig E. Wheelock[1,2,7*,‡]

[1] Gunma University Initiative for Advanced Research (GIAR), Gunma University, 3-39-22 Showa-machi, Maebashi, Gunma, 371-8511, Japan

[2] Division of Physiological Chemistry 2, Department of Medical Biochemistry and Biophysics, Karolinska Institutet, Biomedicum Quartier 9A, Stockholm, 171-77, Sweden

[3] Department of Respiratory Medicine, Amsterdam UMC, University of Amsterdam, Meibergdreef 9, Amsterdam, 1105 AZ, The Netherlands

[4] Department of Experimental Immunology, Amsterdam UMC, University of Amsterdam, Meibergdreef 9, Amsterdam, 1105 AZ, The Netherlands

[5] Computational Physiology and Biostatistics, University Children's Hospital, Spitalstrasse 33, Basel, 4056, Switzerland

[6] Respiratory Medicine Unit, K2 Department of Medicine Solna and Center for Molecular Medicine, Karolinska Institutet, Stockholm, 141-86, Sweden

[7] Department of Respiratory Medicine and Allergy, Karolinska University Hospital, Stockholm, 141-86, Sweden

[8] Department of Biochemistry, Gunma University Graduate School of Medicine, Maebashi, 3-39-22 Showa-machi, Gunma, 371-8511, Japan

†Current address: Key Laboratory of Drug Quality Control and Pharmacovigilance (Ministry of Education), State Key Laboratory of Natural Medicine, China Pharmaceutical University, Nanjing 210009, China

***Corresponding Authors:**
Romanas Chaleckis
Gunma University Initiative for Advanced Research (GIAR),
Gunma University,
Maebashi, Gunma 371-8511, Japan.
Email: romcha@gunma-u.ac.jp .

Craig E Wheelock
Division of Physiological Chemistry 2,
Department of Medical Biochemistry and Biophysics,
Karolinska Institute,
Stockholm 171-77, Sweden.
Email: craig.wheelock@ki.se

‡These authors contributed equally

Table of contents

Experimental section S3

Results and Discussion S4

References S5

Figure S1. Analytical batch layout and sample organization on the 96-well plate S6

Figure S2. Effects of urine normalization upon the multivariate data structure S7

Figure S3. Bland-Altman plots of inter-laboratory refractometry specific gravity readings and freeze-thaw cycle consistency S8

Figure S4. Bland-Altman plots of refractometry-based specific gravity readings (model UG-alpha) with refractive index detector-based specific gravity readings........ S9

Figure S5. Overlaid chromatograms of the technical internal standards in the small study samples and CVs of the corresponding peak areas........ S10

Figure S6. Stability of the ZHP platform annotated metabolites across the reported workflow S11

Figure S7. Stability of the ZHN platform annotated metabolites across the reported workflow S12

Figure S8. Effect of the co-eluting antibiotic trimethoprim on the intensities of the technical internal standard CHES........ S13

Figure S9. Creatinine molecular ion and reporter ion intensities across the injection sequence and extracted chromatograms of the molecular ion and reported ion........ S14

EXPERIMENTAL SECTION

Technical internal standards (tISs)

The tIS concentrations were optimized to achieve intensities 100-300-fold higher than the system noise level (1×10^3) and between 10-30-fold lower than the saturation level in the LC-MS system (3×10^6).

Urine normalization

The urine normalization protocol specifies that all samples are normalized to a value of SG=1.002, which requires dilution with water for the vast majority of samples. The initial volume limitation in preparing the normalized urine is that the raw urine plates contain 105 µL per well following thawing and centrifugation. We have observed that the liquid handler system (Bravo) has reduced precision when transferring volumes <20 µL. We therefore set a final volume of normalized urine equal to 340 µL per well to ensure that the lowest raw urine volume transfer is >20 µL. Accordingly, if the raw urine has a SG >1.006 (1.007-1.030), then a volume of 22.6-97.0 µL of urine is transferred to each well for normalization to give 340 µL per well. However, very dilute raw urine (SG 1.002-1.006) will need to be normalized to a smaller final volume due to the restriction in the available raw urine volume (maximum 100 µL available in the well given a dead volume of <5µL). We suggest that for samples with SG 1.004-1.006, the final normalized volume is reduced to 180 µL. This will require transferring 60-90 µL of raw urine to each well. For samples with SG <1.004, we recommend to use a final volume of 100 µL, necessitating a transfer of 66-100 µL of raw urine to each well. The frequency of urine with low SG is study dependent, but is generally low. Studies using first-morning urine rarely have samples with SG <1.007 (the small study used in this manuscript consisted of morning urine and only 1 individual had SG<1.007). However, the large 842-sample study did not restrict sampling to morning urine only and we observed that ~15% of samples had SG <1.007. Note, the method assumes that the maximum SG value is 1.030, which is to be expected based upon normal physiology. Exceptions to this value would be in cases of bacterial contamination, contrast agent for radiography,[1] very sweet diabetic urine, etc.[2], which biases SG readings and would most likely require sample exclusion.

Data quality check

The quality of the dataset was assessed by examining the following parameters: binary and isopump pressures, overlaid total ion chromatograms (TICs) for all samples and for SQCs only, EICs of lockmasses for all samples and for SQCs only, 3D views (x=RT, y=intensity, z=*m/z*) of one SQC sample and the reference blank sample, EICs of each tIS and calculations of their CVs in the SQCs and in the samples using R scripts. Raw data was first converted to mzML format using Proteo Wizard[3] before loading into MZmine.[4] Extracted ion chromatograms (EICs) of the lockmasses (Table S5) were obtained with 20 ppm tolerance. Targeted peak detection (Table S6) was used for 5 tIS and 25 metabolites (Table S7) that were selected for being common urine metabolites at high intensities, representing different chemical classes and eluting across the whole retention time span. Recommended acceptance criteria for the analysis sequence are: binary pump pressure profile drift <10 bar across acquisition, presence of the lockmass signals above threshold in all spectra, baseline overlay of TICs for all the samples, excellent overlay of TICs in the SQC samples (baseline overlay without retention time shifts or outlier peaks) and tIS EICs in both samples and QCs, 3D views without signs of mobile phase contamination (unusual shark fins) and CVs of tIS ≤10% in the SQCs and ≤20% in the samples. Failure to meet these criteria does not necessarily lead to the rejection of the run, but rather flags the plate for detailed

troubleshooting and careful evaluation as to whether the identified problem compromises the run quality.

Data pre-processing

Unidentified peaks, especially features among the 500 most intense peaks, were tentatively annotated using AM (<10 mDa tolerance) corresponding to MSI level 3. Supporting information for the annotation of the metabolites was a match with in silico fragmentation patterns in MS-FINDER, correlation with related compounds in the dataset, previous reporting of the metabolite in urine, RT relative to structurally similar known compounds, presence of specific fragments, etc.

All the annotated metabolite peak alignments and integrations were individually curated before exporting as a text file. Peak area and height intensities were plotted across the injection sequence for visual inspection using R ggplot2 scripts.[5] As one metabolite can produce several signals (fragments or adducts), the annotated peak list was curated to have only one signal per metabolite. The molecular ion ($[M+H]^+$ or $[M-H]^-$) was generally preferred for choosing the representative molecular species; however, in some cases, a fragment or adduct displaying marked improved analytical performances was selected instead (*e.g.,* creatinine Fig. S9).

RESULTS AND DISCUSSION

Sample preparation

The thawed urine samples are not centrifuged prior to aliquoting. This decision was made based upon the fact that there is no general harmonization on the selection of urine collection tubes. Accordingly, tubes are provided from clinical collaborators in various brands and shapes, and often in larger containers that do not fit available rotors of the centrifuges. To avoid time-consuming manual transfers to centrifugeable tubes, we opted to move the urine clean-up centrifugation step to after aliquoting. In addition, because aliquot plates are frozen at -80°C, thawing will release debris that must be removed. Given that the original samples are not centrifuged prior to transfer to 96-well plates, the amount of particulates between the aliquots may vary; a centrifugation step after thawing is therefore necessary to ensure aliquot homogeneity.

Analytical methods

The ZHP method was optimized from the published version in terms of injection volume and ionization settings. The injection volume was decreased from 2 µL to 0.8 µL to reduce saturation of highly abundant compounds (*e.g.,* creatinine). Saturation can result in a mass shift that may translate into split chromatograms, and interfere with the alignment of untargeted data resulting in artifact peaks and low-quality data.

The ZHN stationary phase was selected after comparison between ZIC-pHILIC and the BEH amide (Waters), the former providing a slightly better performance for acidic amino acids, sugars and keto-acids, while the latter provided a better retention of steroids. We chose the ZIC-pHILIC with the objective of providing the widest chemical coverage. In addition, while the relatively large particle size of the ZIC-pHILIC column (results in broader peaks) and maximum pressure limitation (200 bar) are limitations, the ZIC-pHILIC column showed a good feature distribution across the RT. Switching the mobile phase A buffer from ammonium carbonate, as in several previous publication that use high pH with ZIC-pHILIC,[6, 7] to ammonium acetate and maintaining a low buffer concentration of 5mM enabled the partial rescue of steroid retention and increased performance for tricarboxylic acids. The

method was then further optimized in terms of gradient and run time. The starting gradient was set to 88% B, which revealed the best compromise between low organic content that would impact peak shapes of carboxylic acids and high organic content that would affect steroid peak shape and retention. An isocratic start did not lead to improvements and increased run length time. Gradient steepness was also optimized to avoid a high level of co-elution while optimizing run time and peak shapes of later eluting compounds, which were further improved with the addition of a steeper sequence at the end of the gradient. Finally, a column wash with 25% B was necessary to avoid long-term column clogging, thus calling for a reequilibration time of 10.5 min to achieve a reproducible baseline.

The pH reproducibility of the ZHN method was evaluated by comparing the pH of mobile A solutions prepared on 4 different days. Values differed by a maximum of 0.08 pH units (9.34-9.42). As basic mobile phases are prone to absorb ambient CO_2, which can consequently affect the pH, we evaluated the pH stability of the ZHN mobile phase A stored at room temperature vs. at 4°C, and did not observe any variation after 7 days. However, care should be taken to have proper mobile phase cap fittings.

Another challenge in the automated workflow is the centrifugation force and duration. While common centrifuges with a fixed angle rotor for 1.5 mL tubes (*e.g.,* Eppendorf 5430) can achieve centrifugation forces >20,000 *g*, plate centrifuges with swing rotors are limited to a maximum of 3000-4000 *g*. This limitation can be partially compensated for with longer centrifugation time. We observed differences in metabolite profiles for centrifugation times between 10 and 40 min, while 40 min and 2 h did not evidence major shifts (data not shown). We therefore opted for 40 min centrifugation time. Although plate centrifuges are not able to offer the same pelleting efficiency as fixed angle rotor centrifuges, the supernatant is sufficiently clean to achieve reproducibility and avoid clogging in the LC system.

REFERENCES

(1) Giasson, J.; Chen, Y. A discrepant urine specific gravity. *Clin Chem* **2012,** *58* (4), 797.
(2) Simerville, J. A.; Maxted, W. C.; Pahira, J. J. Urinalysis: a comprehensive review. *Am Fam Physician* **2005,** *71* (6), 1153-62.
(3) Chambers, M. C.; Maclean, B.; Burke, R.; Amodei, D.; Ruderman, D. L.; Neumann, S.; Gatto, L.; Fischer, B.; Pratt, B.; Egertson, J., et al. A cross-platform toolkit for mass spectrometry and proteomics. *Nat Biotechnol* **2012,** *30* (10), 918-20.
(4) Pluskal, T.; Castillo, S.; Villar-Briones, A.; Oresic, M. MZmine 2: Modular framework for processing, visualizing, and analyzing mass spectrometry-based molecular profile data. *Bmc Bioinformatics* **2010,** *11*.
(5) R Development Core Team *R: A language and environment for statistical computing*, Viena, Austria, 2010.
(6) Gallart-Ayala, H.; Konz, I.; Mehl, F.; Teav, T.; Oikonomidi, A.; Peyratout, G.; van der Velpen, V.; Popp, J.; Ivanisevic, J. A global HILIC-MS approach to measure polar human cerebrospinal fluid metabolome: Exploring gender-associated variation in a cohort of elderly cognitively healthy subjects. *Anal Chim Acta* **2018,** *1037*, 327-337.
(7) Zhang, T.; Creek, D. J.; Barrett, M. P.; Blackburn, G.; Watson, D. G. Evaluation of coupling reversed phase, aqueous normal phase, and hydrophilic interaction liquid chromatography with Orbitrap mass spectrometry for metabolomic studies of human urine. *Anal Chem* **2012,** *84* (4), 1994-2001.

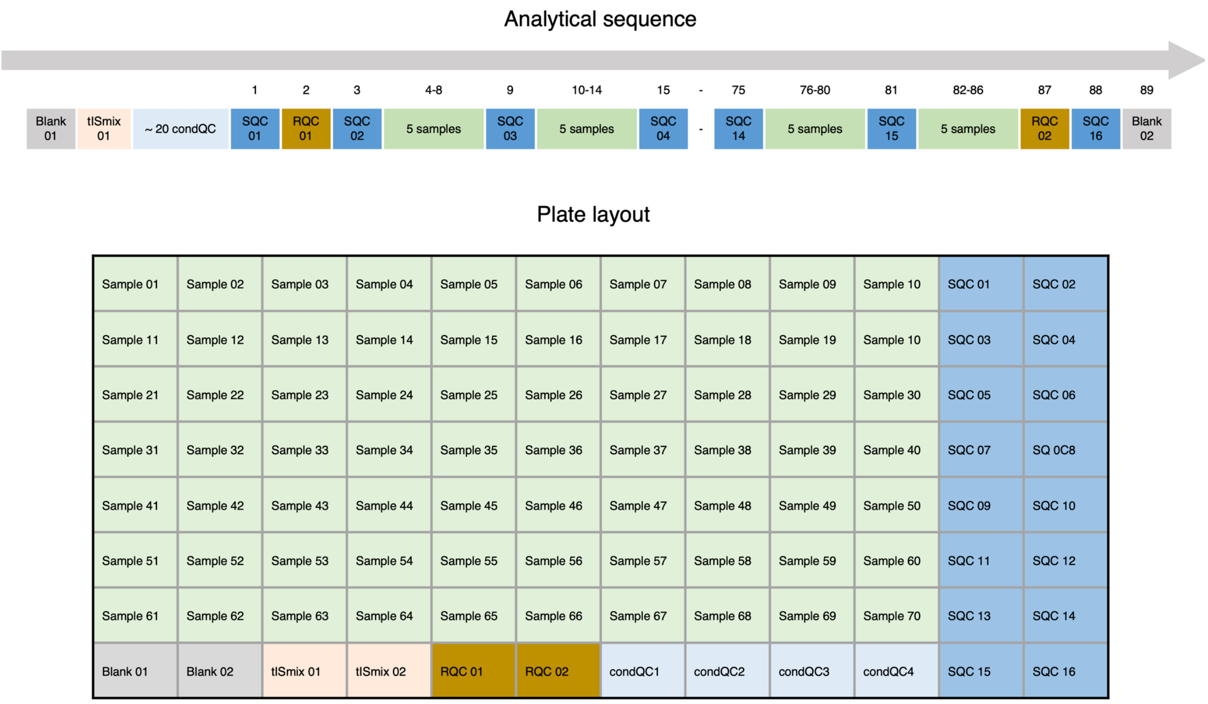

Figure S1. Analytical batch layout and sample organization on the 96-well plate. SQC=pooled study quality control sample (pooled mix of all study samples); condQC=quality control samples for column conditioning (usually additional SQC samples); RQC=reference quality control sample to be included in all studies (prepared from laboratory reference urine); tISmix=technical internal standard mix.

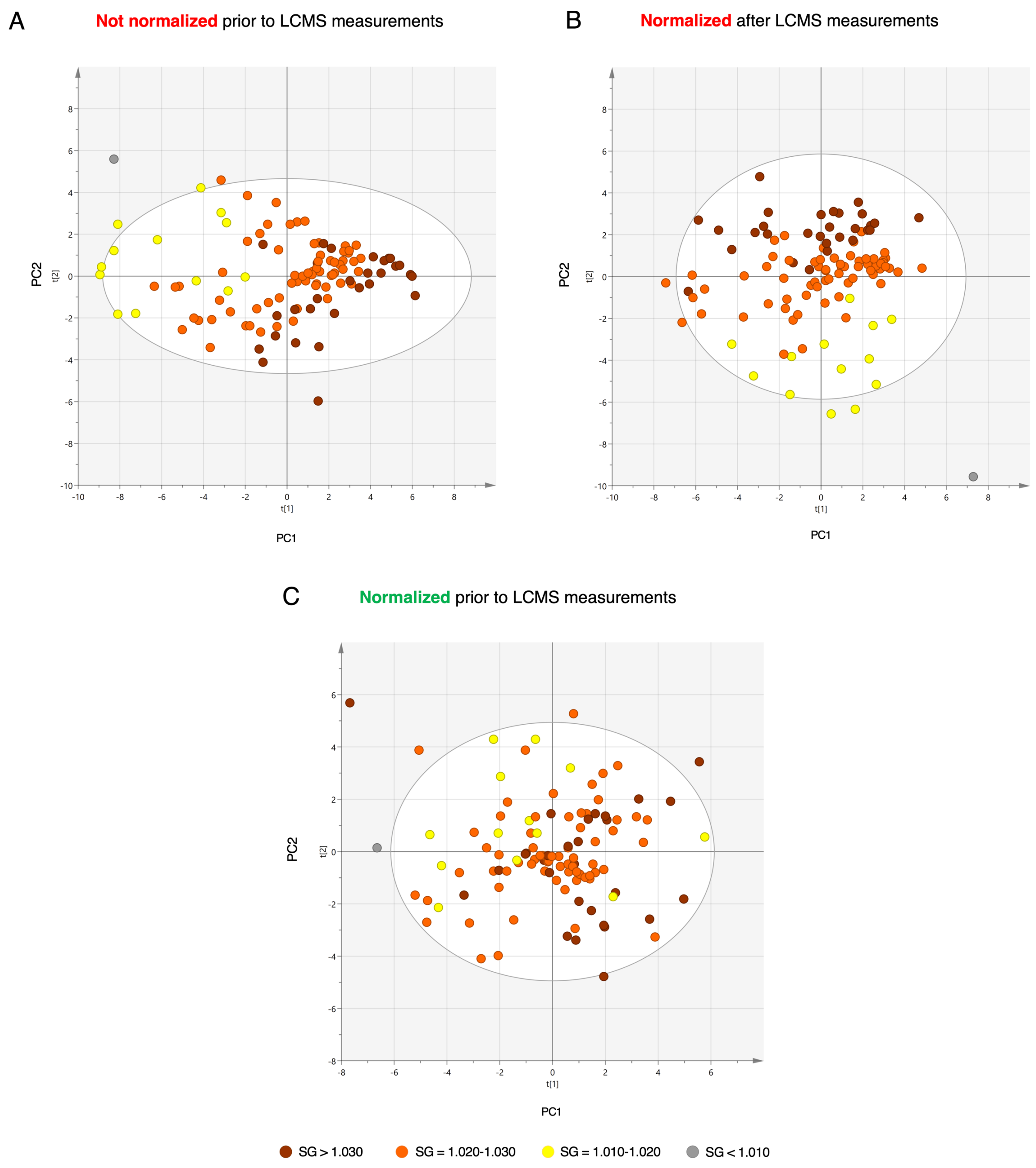

Figure S2. Effects of urine normalization upon the multivariate data structure. Principal components analysis (PCA) scores plots of the 87-sample LUNAPRE cohort measured A) without any normalization prior to acquisition of LC-MS data, B) urinary normalization post-acquisition of LC-MS data, and C) urinary normalization pre-acquisition of LC-MS data. Normalization was performed using the measured urinary specific gravity (SG) of each sample. LC-MS data were acquired using the methods described in the main text. Metabolites were from the data quality check list (30 metabolites) and were log transformed and UV scaled for inclusion in the PCA model. PCA plots were generated using SIMCA v15 (Satorius AG, Göttingen, German). PCA model metrics are as follows: (A) $R^2X=0.599$, $Q^2=0.442$; (B) $R^2X=0.547$, $Q^2=0.341$; (C) $R^2X=0.332$, $Q^2=0.166$.

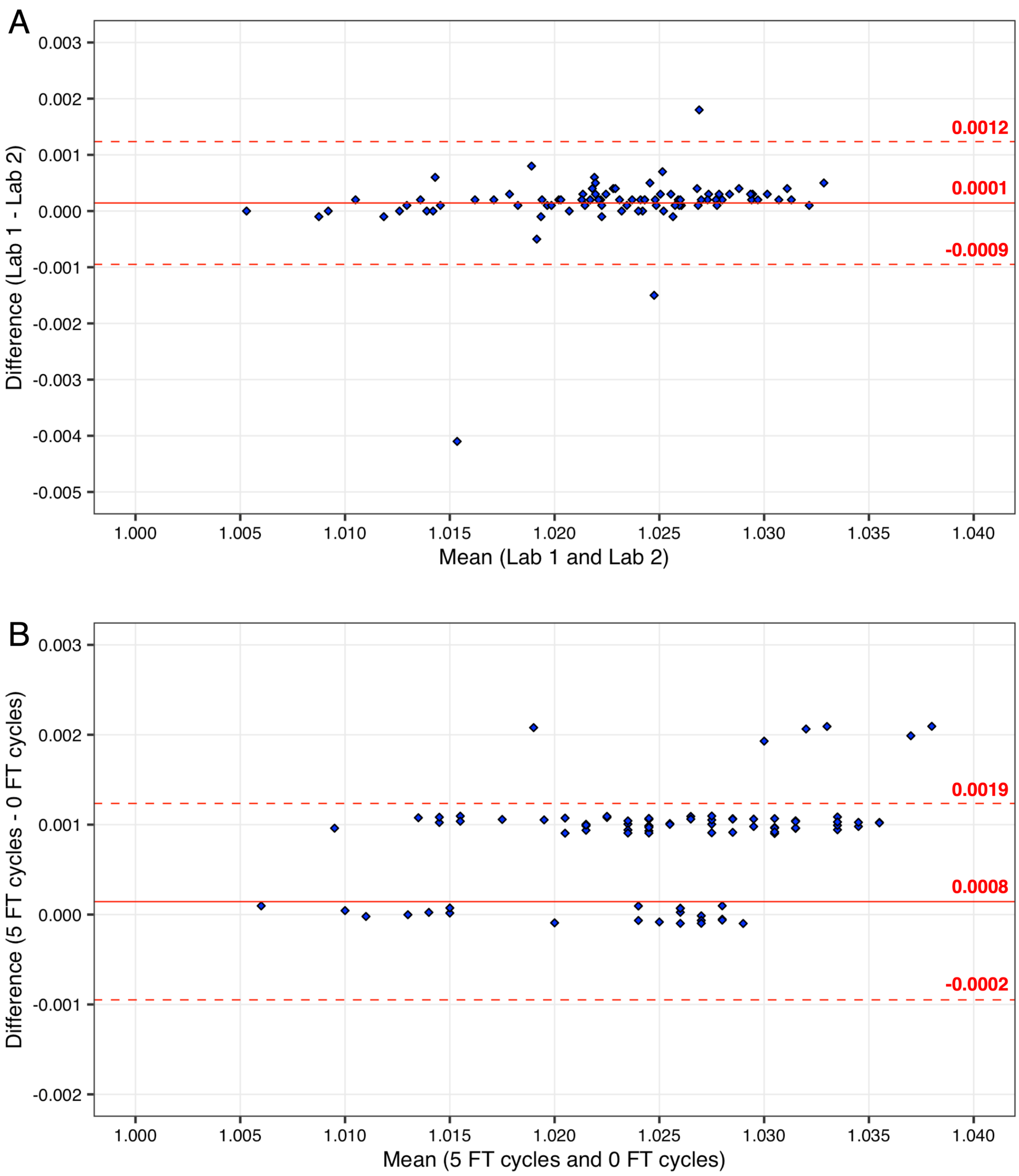

Figure S3. Bland-Altman plots of inter-laboratory refractometry specific gravity (SG) readings (A) and freeze-thaw cycle consistency (B). Each sample is represented by a blue lozenge (n=87), mean deviation as a solid red line, and 95% confidence intervals (limits of agreement, LOA) with red dotted lines. A) SG readings from the refractometer model UG-alpha between 2 different laboratories (Lab 1=Japan, Lab 2=Sweden). B) SG readings from the refractometer model UG-D of samples following 5 additional freeze-thaw cycles compared to controls.

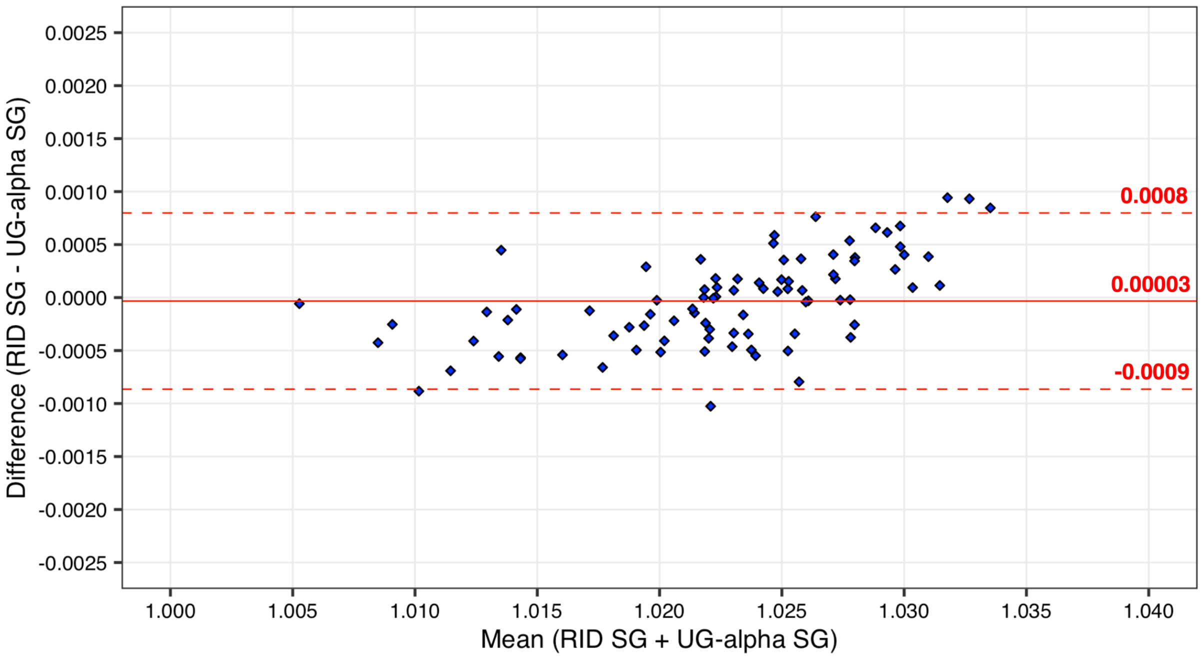

Figure S4. Bland-Altman plots of refractometry-based specific gravity (SG) readings (model UG-alpha) with refractive index detector-based SG readings (n=87). Each sample is represented by a blue lozenge, mean deviation as a solid red line, and 95% confidence intervals (limits of agreement, LOA) with red dotted lines. SG measurements using the Agilent refractive index detector (RID) are described in the main text.

ZHP platform	Pyrantel	CHES	5-Fluorocytosine	PIPES	HEPES
Chromatography	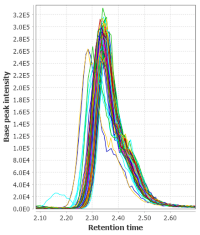	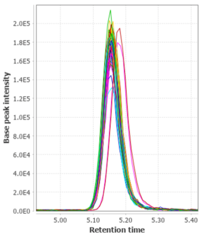	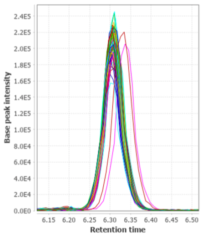	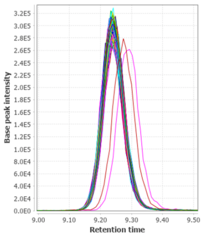	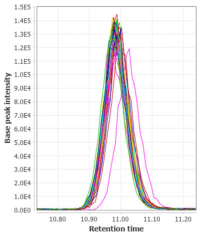
CV (QC/sample)*	3.0 / 2.5	3.7 / 6.2	3.8 / 7.0	4.0 / 3.5	4.4 / 3.1
**ZHN platform**	**4-Fluorocinnamic acid**	**CHES**	**HEPES**	**PIPES**	**Tricarballylic acid**
Chromatography	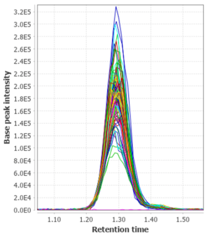	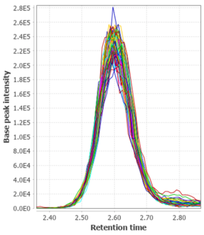	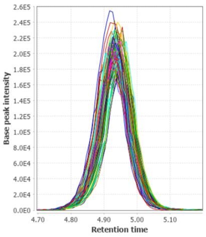	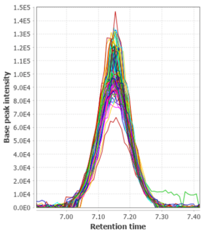	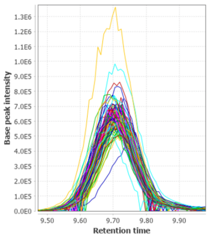
CV (QC/sample)*	10.0 / 20.0	4.1 / 6.3	7.5 / 8.2	5.7 / 10.0	5.6 / 18.0

Figure S5. Overlaid chromatograms of the technical internal standards (tIS) in the small study samples and CVs of the corresponding peak areas. The CVs are shown from both the primary small study samples (n=87) as well as the pooled study quality control (SQC) samples (n=22). ZHP=ZIC-HILIC positive ionization; ZHN=ZIC-pHILIC negative ionization.

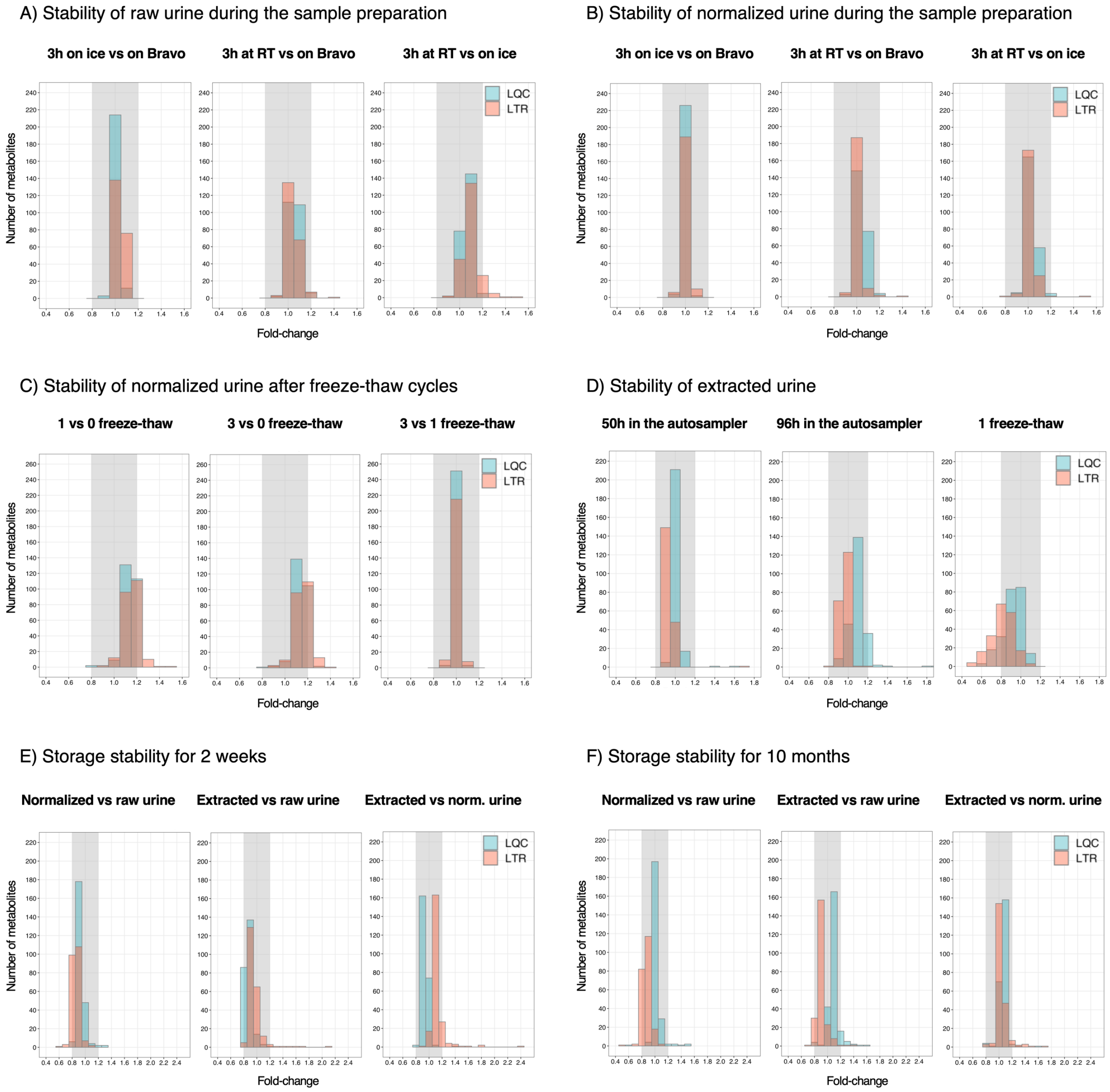

Figure S6. Stability of the ZHP platform annotated metabolites across the reported workflow. A) The temperature-dependent stability of the observed metabolites in raw urine during the sample preparation. B) The temperature-dependent stability of the observed metabolites in normalized urine during the sample preparation. C) The effect of multiple freeze-thaw cycles on metabolite stability in normalized urine. D) Stability of metabolites in extracted urine for up to 96-hours. E) Stability of normalized, extracted, and raw urine for 2 weeks at -80°C. F) Stability of normalized, extracted, and raw urine for 10 months at -80°C. Metabolite annotations (in semi-transparent red, metabolites from the SQC urine; in blue from the RQC) are from the annotated dataset (Fig. 4A in the main text) and acquired by LC-MS as described in the main text. ZHP=ZIC-HILIC positive ionization; RT=room temperature; RQC=reference quality control; SQC=pooled study quality control; Bravo=automated liquid sample handler (Agilent Technologies).

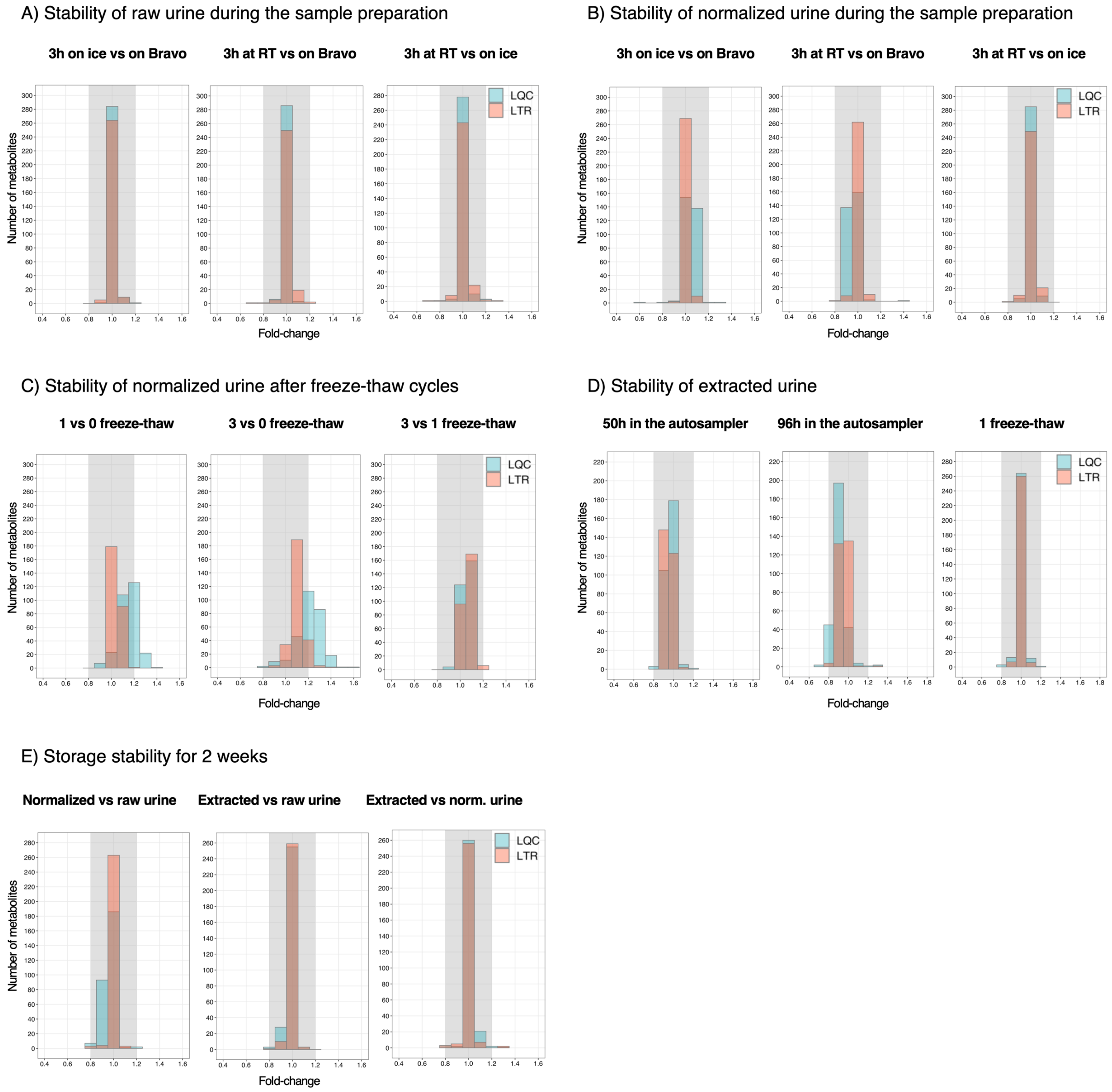

Figure S7. Stability of the ZHN platform annotated metabolites across the reported workflow. A) The temperature-dependent stability of the observed metabolites in raw urine during the sample preparation. B) The temperature-dependent stability of the observed metabolites in normalized urine during the sample preparation. C) The effect of multiple freeze-thaw cycles on metabolite stability in normalized urine. D) Stability of metabolites in extracted urine for up to 96-hours. E) Stability of normalized, extracted, and raw urine for 2 weeks at -80°C. Metabolite annotations (in semi-transparent red, metabolites from the SQC urine; in blue from the RQC) are from the annotated dataset (Fig. 4A in the main text) and acquired by LC-MS as described in the main text. ZHN=ZIC-HILIC negative ionization; RT=room temperature; RQC=reference quality control; SQC=pooled study quality control; Bravo=automated liquid sample handler (Agilent Technologies).

Raw data (per plate)

ZHP	Mean CV_{QC} (min-max)	Mean CV_{sample} (min-max)
Pyrantel	2.7 (1.0 - 4.8)	3.7 (1.5 - 6.8)
CHES	3.6 (1.4 - 6.9)	6.8 (4.1 -16.0)
Fluorocytosine	8.5 (2.3 - 19.0)	16.0 (5.5 - 31.0)
PIPES	4.4 (1.7 - 9.2)	6.7 (3.1 - 13.0)
HEPES	4.5 (1.5 - 9.2)	6.2 (1.9 -15.0)

CHES intensities in plate 11 (large cohort)

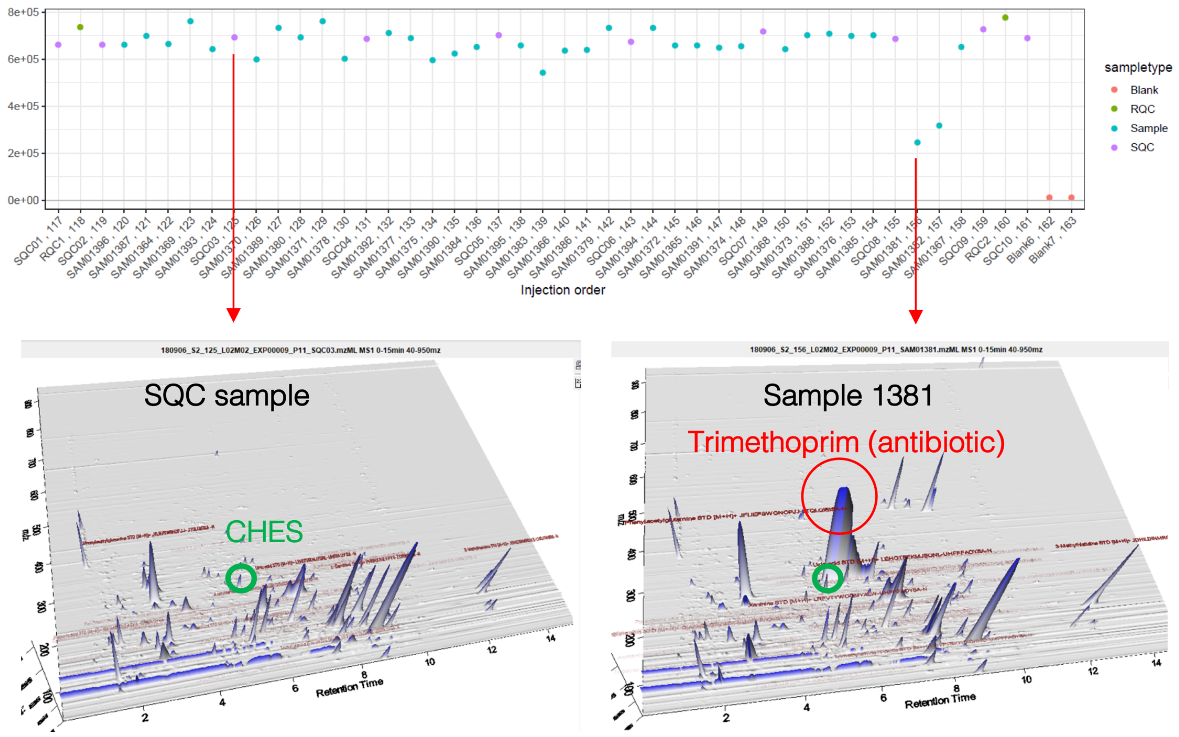

Figure S8. Effect of the co-eluting antibiotic trimethoprim on the intensities of the technical internal standard (tIS) CHES. ZHP=ZIC-HILIC positive ionization; SQC=pooled study quality control.

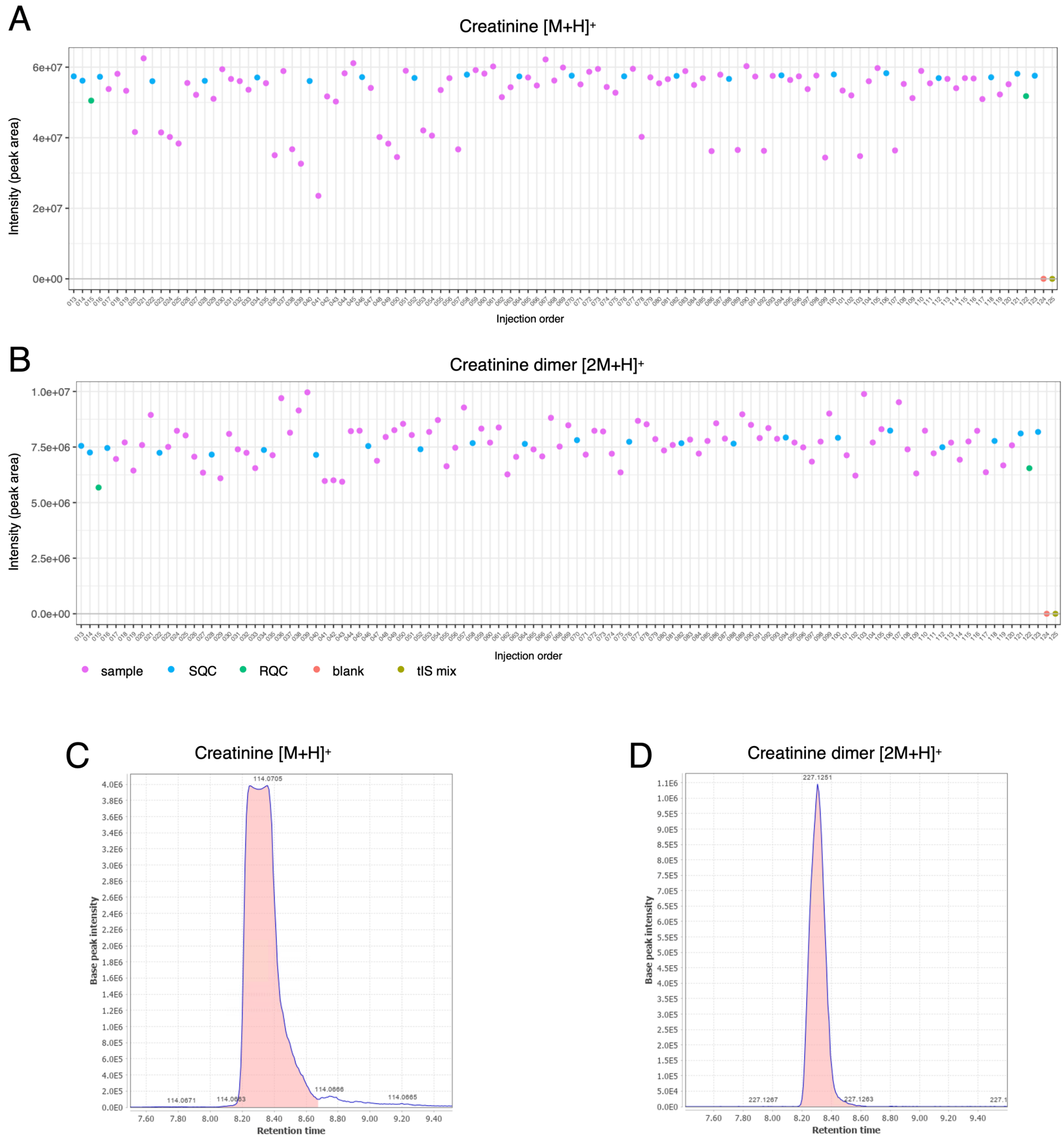

Figure S9. Creatinine molecular ion (A) and reporter ion (B, dimer) intensities across the injection sequence and extracted chromatograms of the molecular ion (C) and reported ion (D, dimer). SQC=pooled study quality control sample; RQC=reference quality control sample; tIS mix=technical internal standard mix.
